# Assessing the sensory and physicochemical impact of reverse osmosis membrane technology to dealcoholize two different beer styles

**DOI:** 10.1016/j.fochx.2021.100121

**Published:** 2021-04-30

**Authors:** Imogen Ramsey, Qian Yang, Ian Fisk, Charfedinne Ayed, Rebecca Ford

**Affiliations:** a Sensory Science Centre, Division of Food, Nutrition and Dietetics, School of Biosciences, University of Nottingham, Sutton Bonington Campus, Loughborough LE12 5RD, United Kingdom; b Food Flavour Laboratory, Division of Food, Nutrition and Dietetics, School of Biosciences, University of Nottingham, Sutton Bonington Campus, Loughborough LE12 5RD, United Kingdom

**Keywords:** Physicochemical, Sensory, Reverse osmosis, Non-alcoholic beer, Dealcoholization

## Abstract

A pilot scale dealcoholisation unit fitted with reverse osmosis (RO) membranes was used to directly compare two beer matrices (stout, lager, ~ 5% ABV) and their dealcoholized counterparts (~0.5% ABV), for physicochemical properties (volatiles, pH, ABV, polyphenols, bitterness) and sensory profiles using a trained descriptive panel (n = 12). The efficiency and consistency of RO membranes were evaluated by replicate dealcoholisation trials (n = 3) for each beer. Statistical analysis revealed significant reductions (p < 0.05) in key volatile compounds with linear structures (ethyl octanoate, octan-1-ol) compared to those with increased levels of branching (3-methylbutyl acetate, 2-methylpropan-1-ol). Significant reductions (p < 0.0001) in ‘fruity/estery’, ‘alcoholic/solvent’, ‘malty’, ‘sweetness’ and ‘body’ sensory attributes were also discovered. Finally, longer processing times for the stout across replicate trials suggested membrane clogging, whilst differences in volatile reduction suggested membrane fouling. This novel research proposes compound structure, rather than compound size, impacts RO membrane permeability and resulting sensory quality.

## Highlights

• Reverse osmosis results in significant losses in volatile compounds and modified sensory profiles.
• Volatile losses appear to be related to compound structure, not compound size.
• Reverse osmosis efficiency varies between beer styles, with longer processing times for stouts.
• Reverse osmosis membranes are susceptible to fouling over time, affecting overall product quality.

## 1 Introduction

Although beer is the most consumed alcoholic beverage in the Americas and Europe (World Health Organisation, 2018), sales have fallen since 2012 by over 150 million litres (Mintel, 2017b). A new revolution of health conscious consumers has been on the upward trend, with a third of UK consumers limiting their alcohol consumption to improve their health, manage weight and reduce the risk of disease (Mintel, 2017a). A rise in sales of non–alcoholic beer (NAB) in European countries such as Spain and Germany has been observed, with output increasing by almost 50% since 2014 (Euromonitor, 2019) and total volume growth in the UK increasing by 29% between 2013 and 2018 (Euromonitor, 2019). Consequently there has been increased interest in the development of NAB, with global manufacturers committing to responsible drinking targets by promising to increase their overall NAB range (ABInBev, 2018). Nevertheless, there is still a way to go in producing a NAB which is sensorially similar to a standard beer, with both consumer studies and market research reports stating that consumers find lower alcohol alternatives to be ‘bland’, ‘disappointing’ and ‘less tasty’ (Chrysochou, 2014, Mintel, 2015). Therefore, more research needs to be conducted to understand the key physicochemical and sensorial losses occurring during NAB production processes so that future research can attempt to improve the quality of NAB to that of a standard alcohol beer, thus increasing consumer liking.

NAB can be produced through numerous methods, which can be categorised into biological or physical processing, however all of these methods will have some effect on the resulting sensory properties of the NAB. Biological processing includes arrested fermentation, use of special yeasts and altered mashing processes (Branyik, Silva, Baszczynski, Lehnert, & Silva, 2012). Physical processing can include thermal processes, such as rectification or thin film evaporation, whereas membrane processes can include dialysis, osmotic distillation, pervaporation, nanofiltration (NF) and reverse osmosis (RO) (Blanco et al., 2016, Branyik et al., 2012, Güzel et al., 2020). In a comprehensive review on NAB production, Branyik et al. (2012) found that all techniques produced significant losses in volatiles, however RO seemed to show the smallest change, with further encouraging results found from other researchers when dealcoholizing beer, wine and cider (Alcantara et al., 2016, Catarino et al., 2007, Catarino, 2010, Gil et al., 2013, Lopez et al., 2002, Pilipovik and Riverol, 2005).

RO therefore appears to be one of the most promising techniques to produce a NAB. To summarise the technique, pressurised beer (20–80 bar) is passed through a semi-permeable membrane (Pilipovik & Riverol, 2005), which is theoretically permeable to low molecular weight molecules such as water and ethanol which are removed from the product into the permeate stream. The membrane is less permeable to larger molecules such as carbohydrates, colours and flavours, which can be fed back into the retentate beer tank (Müller, Bellut, Tippmann, & Becker, 2017). RO can be operated at low temperatures and pressures, reducing energy consumption with limited flavour losses (Catarino et al., 2007). As there are large water losses during processing, water needs to be added back in via diafiltration, which can be described as continuous (adding water back in during processing at different time points) or discontinuous (by diluting the product at the beginning or rediluting at the end to its original starting volume) (Branyik et al., 2012). The final product is then recarbonated as CO₂ is lost during the process (Hodenberg, 1991). Membranes can be made from cellulose acetate, polyamide or polyimide on polyester, polysulfone, or fibreglass support structures (Branyik et al., 2012) and are normally placed in geometric arrangement modules which can include planar, tubular or spiral-wound (Light, Mooney, Chu, & Wood, 1986). It has been reported, that the minimum achievable alcohol content is around 0.5% ABV due to economic feasibility (Catarino et al., 2007, Pilipovik and Riverol, 2005). However, in a recently published study by Ramsey, Yang, Fisk, and Ford (2021) investigating physicochemical differences between commercially produced non-alcoholic lagers, it was discovered that commercial beers produced using physical dealcoholisation techniques in general had the lowest ABV (0.05–0.08% ABV) compared to other production methods. This therefore shows the advancements in technologies in this sector, as well as the improved economic feasibility of this technique. Nevertheless, knowledge of flavour and sensorial differences in the production of dealcoholized beer through RO is very limited, with little published data understanding the permeability of compounds from different beer matrices and the resulting impact on sensory perception.

Previous studies conducted using RO have mainly focused on improving efficiency, by reporting on different operating parameters (pressure, temperature, membrane materials, and operating modes) with various alcoholic beverages (Catarino et al., 2006, Catarino et al., 2007, Falkenberg, 2014, Lopez et al., 2002, Pilipovik and Riverol, 2005). However, to date only one study has investigated membrane efficiency between replicate trials (Falkenberg, 2014), reporting differences in ethanol reduction timings between subsequent runs using the same membranes. This was proposed to be due to soiling or fouling of the membranes (Falkenberg, 2014). Understanding membrane capabilities, as well as the potential changes in finished product quality, is important for breweries to understand, especially if membranes are prone to soiling or fouling. Changes between trials can have a significant effect on the overall product quality for consumers, and therefore these differences need to be addressed.

Research exploring differences between a standard beer and its RO dealcoholized counterpart have focused on key brewing parameters (colour, bitterness, pH, alcohol content) (Alcantara et al., 2016), or volatile profiles (using headspace solid phase microextraction gas chromatography (HS-SPME-GC–MS)) (Riu-Aumatell, Miro, Serra-Cayuela, Buxaderas, & Lopez-Tamames, 2014). Only one study combined HS-GC–MS techniques with sensory data, to assess the differences between lagers produced by different membrane filtration techniques (RO and NF), comparing them back to the original 5% beer (Falkenberg, 2014). However, sensory analysis was not conducted using typical ISO standards, and should therefore be interpreted with caution. To the authors’ knowledge no studies have directly compared the sensory and physicochemical impact of RO on different beer styles. It is hypothesised here that a difference in the starting matrix through the use of different raw materials may have an effect on the membrane efficiency, resulting in changes to the physicochemical and sensory properties of the resulting NABs. This is valuable information for brewers to understand if the same RO equipment could potentially be used to develop a range of NAB styles to satisfy a variety of consumer needs.

Therefore, in the present study, the impact of RO on the physicochemical and sensory properties of different beer styles was assessed to understand the efficacy of this method for producing lower alcohol versions of standard beers. The objectives of this study were to explore the use of dealcoholization using RO membranes on i) the key physicochemical and sensorial properties of two different beer styles compared to their standard strength equivalents; ii) the influence of compound characteristics (molecular weight, Log*P* and structure) on their removal; iii) matrix-membrane interactions; iv) membrane efficiency by performing replicate trials. It is hoped that results from this study will help improve RO techniques in the production of NAB in future research, by proposing a mechanism for specific compound removal and indicating the resulting sensory changes.

## 2 Materials and methods

### 2.1 Beer samples

A lager and stout were purchased (300 L each) from a local brewery (same overall batch) and were delivered as 6 × 50 L kegs (East Sussex, UK). The lager was a 5.1% ABV Pilsner with the following ingredients: pale lager and cara pils malt, Mittlefruh leaf hops and Saaz leaf hops, SafLager W-43/70 yeast. The stout was a 4.3% ABV oatmeal stout with the following ingredients: pale ale, chocolate, wheat, light crystal and Carafa Spieziel III malts, flaked oats, roasted barley, Fuggles hops and SafAle US-05 yeast.

### 2.2 Dealcoholization

**Fig. 1:**
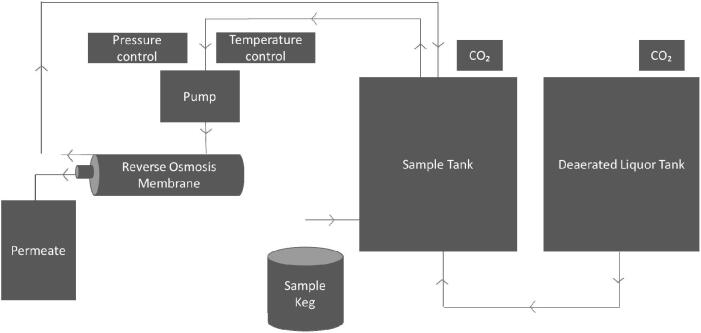
Reverse osmosis dealcoholization set up in a closed system for trials.

Dealcoholization tests were conducted using a pilot-scale LabStak M20-0.72 unit (Alfa Laval, Lund, Sweden) fitted with an RO90 spiral wound membrane made up of a thin film composite polyamide membrane with polyester support material, measuring 1.9 m^2^ (Alfa Laval, Lund, Sweden). For all trials, modifications were made to ensure that the M20-0.72 unit was operated in a ‘closed’ environment, pressurised with CO₂ and suitable for use within a commercial setting. The cross flow rate of the module was 8 L/min. For each replicate trial performed, 50 L beer was introduced from the purchased commercial keg into the sample tank, which had a maximum capacity of 160 L. The unit was then turned on, allowing the pump to process beer from the sample tank through the membrane. Ethanol and water were removed through the permeate tube and dealcoholized beer was processed back into the sample tank to be dealcoholized again. This process was performed on a continuous loop until the beer reached its desired ethanol concentration. At regular time points deaerated brewing liquor, pressurised with CO₂ to avoid oxygen problems, was added back into the sample tank following continuous diafiltration. Previous trials confirmed the selection of membrane type to be used (RO90), operating temperature (20 °C) and trans-membrane pressure (20 bar) by calculating ethanol reduction efficiency, least volatile reduction and economic viability. Temperature was controlled before entering the membrane module, with cooling water used as a cooling medium for the sample and deaerated brewing liquor tanks. Pressure was controlled using the RO pressure dial. A basic diagram of the set-up is shown in Fig. 1. Before starting dealcoholization, 3 × 50 L kegs of each standard beer style were labelled as S1, S2 and S3 and were transferred into 275 mL bottles. All dealcoholization trials were performed in triplicate to understand the efficiency of the membrane, with beer transferred into 275 mL bottles and labelled according to the replicate trial: D1, D2 and D3.

#### 2.2.1 Membrane cleaning

Cleaning followed manufacturers’ membrane cleaning guidelines, by flowing mains water through the system for 20 min. Subsequently, a 0.1% NaOH solution at 30–40 °C was circulated for 20 min and then rinsed with mains water again for 20 min. This procedure was completed after every trial.

### 2.3 Physicochemical analysis

Ethanol content was measured using an Anton Paar Alcolyzer and DMA4500 (Graz, Austria). Sample pH was determined using a Metler Toledo FiveGo pH meter (Colombus, Ohio, USA) after calibration with pH 4.0 and 7.0 standards. Total polyphenol (TP) content and bitterness units (BU) were determined using the international methods by the American Society of Brewing Chemists (ASBC) (Beer-35, Beer-25A respectively) (ASBC Method of Analysis, 2015, ASBC Method of Analysis, 2018).

Headspace Gas Chromatography- Flame Ionization Detector (HS-GC-FID) lower boiling point beer volatile analysis was determined using the method by Analytica-European Brewing Convention (EBC) (9.39) (Analytica-EBC, 2018). Beer samples (10 mL) were transferred into glass vials with 3.5 g sodium chloride and 50 µL 1-butanol (internal standard at final concentration of 50 mg/L). Volatiles were analysed with a Scion 456-Gas Chromatograph (Scion Instruments, West Lothian, UK). Samples (500 µL) were incubated at 60 °C for 20 min with shaking, and then were injected in splitless mode using a PAL Combi-XT autosampler (PAL System, Zwingen, Switzerland) onto a Zebron ZBWax column (60 m × 0.25 ID; Phenomenex Inc, Cheshire, UK). Column temperature was held initially at 85 °C for 10 min, increased by 25 °C/min to 110 °C, before finally being increased by 8 °C/min to 200 °C. Total run time was 36.25 min. The GC carrier gas was helium, at a constant pressure of 15 psi. Full scan mode was used to detect volatile compounds (mass range from *m*/*z* 35 to 200). Volatile compounds were identified by their *m*/*z*, and quantified with the use of six-point calibration curves generated from pure and internal standards. The following aroma compounds were purchased from Sigma-Aldrich (UK) for standard identification: acetaldehyde (≥99.5%), ethyl acetate (≥99.5%), isobutyl acetate (2-methylpropyl ethanonate) (≥97%), propan-1-ol (≥99%), isoamyl acetate (3-methylbutyl acetate) (≥97%), 3-methyl-1-butanol (≥99%), ethyl octanoate (≥98%) and ethyl decanoate (≥98%). Other compounds were purchased from Thermo Fisher Scientific (UK): 1-butanol (≥99.5%), ethyl butanoate (≥99%), 2-methylpropan-1-ol (≥99%) and ethyl hexanoate (≥99%).

To detect other relevant volatile compounds not found through HS-GC-FID analysis, SPME and liquid extraction (LE) were used. For SPME-GC-MS analysis, beer samples (5 mL) were transferred into glass vials and 100 µL 3-heptanone (internal standard at a final concentration of 100 µg/L) was added, and analysed using a modified published method by Yang et al. (2016). Modifications to the method included incubation of samples at 40 °C for 2 min with shaking, with volatile aroma compounds extracted for 10 min and desorped for 1 min. Samples were injected in splitless mode using a PAL Combi-XT autosampler (PAL System, Zwingen, Switzerland) onto a Zebron ZBWax column (30 m × 0.25 ID; Phenomenex Inc, Cheshire, UK). Column temperature was held initially at 40 °C for 2 min, increased by 8 °C/min to 240 °C and held for 1 min. Total run time was 38 min. For LE-GC-MS analysis, beer samples (20 mL) were transferred into a 50 mL conical-based glass tube with 2 mL dichloromethane (DCM) and 100 µL 3-heptanone (internal standard at a final concentration of 25 µg/L), using a modified published method by Holmes, Hense, Donnelly, and Cook (2014). The tube was sealed with a PTFE lined cap and placed on a roller bed at room temperature (150 rpm for 1 h). After extraction, samples were centrifuged at 1000 rpm (123 ×*g*) for 2 min and then the DCM layer was transferred into a glass vial ready for analysis. DCM extracts were analysed with a Scion 456-Gas Chromatograph (Scion Instruments, West Lothian, UK). Samples (1 µL) were injected in splitless mode using a PAL Combi-XT autosampler (PAL System, Zwingen, Switzerland) onto a Zebron ZBWax column (60 m × 0.25 ID; Phenomenex Inc, Cheshire, UK). The GC carrier gas was helium, at a constant pressure of 18 psi. Column temperature was held initially at 40 °C, and then increased by 6 °C/min to 225 °C. Full scan mode was used to detect volatile compounds for both SPME and LE methods (mass range from *m*/*z* 35 to 200). Volatiles were identified by their *m*/*z* and comparison of each mass spectrum with either the spectra from authentic compounds or with spectra in reference libraries (NIST/EPA/NIH Mass Spectral Library, version 2.0, Faircom Corporation, U.S). The quantification of volatiles was expressed by the peak area ratio (PAR), which was calculated by the GC peak area for the compound divided by the peak area of the internal standard.

### 2.4 Sensory analysis

The sensory attributes of the lager and stout samples were evaluated by trained beer panellists (n = 12) from the Campden BRI beer panel using a modified quantitative descriptive analysis (QDA) approach (Stone & Sidel, 2004). Informed consent was obtained for the experimentation with panellists. Assessors had a minimum of 100 h experience in generic descriptive analysis of beer samples. Panel monitoring and training occurred through participation in LGC Standards Proficiency Testing (Teddington, Middlesex, UK) Brewing Analytes-Chemistry (BAPS-CHEM) Level 5 Sensory. Panellists also received monthly refresher training sessions with attributes, definitions and reference standards (data not shown, Appendix Table 1), to assess their ability to describe, discriminate and replicate. All attributes were evaluated using a continuous unstructured line scale, with marks converted to a score of ten for data analysis purposes.

Final sample evaluation was carried out at the Campden BRI sensory facility (Nutfield, Surrey, UK) conforming to ISO standards (ISO 8589: 2007) and included three sessions for each beer style, allowing for triplicate evaluation of each sample by each panellist. Beer samples (50 mL), labelled with three-digit codes, were served at 12 ± 2 °C in lidded black glasses under red light in a balanced, blocked and randomised presentation order. A maximum of six samples were evaluated per two-hour session, with a 10 min break after every two samples, to ensure no carryover or fatigue effects. Panellists were instructed to assess each sample for aroma, taste and mouthfeel attributes using Compusense cloud™ (Guelph, Canada) and were told to expectorate the sample after evaluating. The order of attributes was agreed with panellists before final evaluation took place, starting with the attribute that was perceived first and ending with the last. Unsalted crackers (Tesco, UK) and filtered water (Brita filter jug) were provided for palate cleansing.

### 2.5 Statistical analysis

Analysis of variance (ANOVA) followed by Tukey’s Honest Significant Difference (HSD) post hoc test were conducted at p < 0.05 for instrumental analysis. To identify the difference between the standard and dealcoholized beer for each trial replicate, the % decrease was calculated. All analyses were conducted in duplicate across three sample bottles from the same batch, and a mean calculated. Compound characteristics (molecular weight, Log*P*, structure) were assessed to understand the impact on their removal by the membrane molecular operating environment (MOE) (2002.03, Chemical Computing Group, Montreal, Canada). Partial least squares regression (PLS-R) analysis was conducted (p < 0.05), to model the relationship between GC–MS analysis for each beer style (dependent variable, X-matrix) and molecular descriptors (independent variable, Y-matrix). GC–MS analysis was calculated as a ratio of the peak area of the standard beer compared to the dealcoholized beer. All mean-centred peak areas were initially subjected to PLS-R for dimensionality reduction, and the molecular descriptors for which Variable Importance in Projection (VIP) values were less than 1 were excluded from further analysis. The remaining descriptors were subjected to a second PLS-R, where the total variance of the dataset was cumulatively explained by a limitless number of variables. The scores of the first three molecular descriptors were extracted and used as key variables in the model parameters (R^2^ > 0.5, Q^2^ > 0.5).

A two factor ANOVA (sample, panellist) with interaction and Tukey’s HSD post hoc test was performed on sensory results.

In order to explore the relationships between physicochemical properties and sensory data for each beer style, a PCA was conducted. Both datasets used averaged scores across samples and only included sensory attributes and compounds which significantly discriminated amongst the samples, assessed by ANOVA. Data analyses were performed using XLSTAT (v19.01, Addinsoft, New York, USA).

## 3 Results

### 3.1 Trials

Details of replicate trials for both lager and stout are shown in Table 1. For all lager dealcoholisation trials (D1, D2, D3) and two stout trials (D1, D2) similar starting volumes and final ethanol concentrations were shown. Unfortunately, however the third replicate (D3) for the stout had a smaller starting volume, due to loss of standard beer when transferring from keg to sample tank, resulting in a lower final ethanol concentration and shorter run time. Permeate flowrate interestingly changed with different beer styles, with a reduction from trial 1 to trial 3 for the lager, but an increase for the stout. Stout run times were significantly higher than for the lager, averaging around 10 h compared to 7 h for the lager.

**Table 1:** Starting product volume, initial and final ethanol concentrations, permeate flowrate and run time of three replicate trials for lager and stout style beers using pilot scale LabStak M20-0.72 unit.

Beer Style	Lager			Stout		
Replicate	D1	D2	D3	D1	D2	D3
Starting Product Volume (L)	50.5	51.2	44.2	51.6	52.4	18.0
Initial Ethanol Concentration (ABV)	5.09	5.09	5.10	4.31	4.29	4.30
Final Ethanol Concentration (ABV)	0.45	0.47	0.40	0.35	0.35	0.08
Permeate Flowrate (mL/min)	420	385	350	230	250	272
Run time	6 h 43mins	7 h 30mins	6 h 30mins	10 h 35mins	9 h 50mins	3 h 03mins

### 3.2 Physicochemical results

Physicochemical analysis for each trial are presented for the lager (Table 2a) and the stout (Table 2b). Results for ethanol concentration showed a significant reduction after dealcoholization for all replicates, with around 91% for all lager trials, 92% for stout D1 and D2 and 98% for D3 - due to a smaller starting volume for stout D3. The results from these trials therefore confirmed that RO is a suitable technique for removing ethanol from beer. pH, bitterness and total polyphenol content were also shown to decrease for all trials, however a small increase in pH was shown for the stout trials. It is unclear as to why this increase occurred. HS-GC-FID analysis allowed the identification and quantification of the most abundant compounds, showing that the concentration of all compounds for both lager and stout in each of the dealcoholization trials were significantly different (p < 0.05) from the starting concentration. Interestingly, a difference amongst replicate trials for each beer style was also shown. For both the lager and the stout, increased removal of higher alcohols (propan-1-ol, 2-methylpropan-1-ol and 3-methyl-1-butanol) was shown for trial D3 in comparison to trials D1 and D2. Esters (ethyl acetate, ethyl butanoate and 3-methylbutyl acetate) also followed a similar trend for the stout.

**Table 2:** Physicochemical results (ABV, pH, Bitterness Units, Total Polyphenols and Lower Boiling Point Volatiles for A) Lager and B) Stout trials 1, 2 and 3). % change was calculated for each trial replicate as a percentage left from the standard beer to the dealcoholized beer. Different letters within a row^abc^ represent a significant difference among samples in terms of volatile concentrations (Tukey’s HSD, p < 0.05).

**A**										
Measurements		Trial 1			Trial 2			Trial 3		
		Standard Beer (S1)	Dealcoholized Beer (D1)	% Change	Standard Beer (S2)	Dealcoholized Beer (D2)	% Change	Standard Beer (S3)	Dealcoholized Beer (D3)	% Change
Ethanol Concentration (ABV)		5.09^a^	0.45^b^	*−91.1*	5.09^a^	0.40^c^	*−92.1*	5.10^a^	0.47^b^	*−90.8*
pH		4.50^a^	4.38^b^	*−2.6*	4.50^a^	4.36^b^	*−3.1*	4.51^a^	4.37^b^	*−3.2*
Bitterness Units		16.32^a^	11.36^c^	*−30.4*	14.93^b^	11.49^c^	*−23.0*	16.16^ab^	10.42^c^	*−35.5*
Total Polyphenols (mg/L)		282.08^a^	239.80^bc^	*−15.0*	262.58^ab^	224.68^c^	*−14.4*	272.42^a^	216.85^c^	*−20.4*
Volatile Compounds (mg/L)	Acetaldehyde	11.90^a^	3.42^c^	*−71.3*	12.24^a^	1.96^c^	*−84.0*	12.09^a^	6.78^b^	*−43.9*
	Ethyl Acetate	45.73^a^	3.83^b^	*−91.6*	39.88^a^	3.46^b^	*−91.3*	46.58^a^	4.75^b^	*−89.8*
	2-Methylpropyl Ethanoate	0.00^b^	0.02^b^	*+100*	0.00^b^	0.01^ab^	*+100*	0.00^b^	0.01^b^	*+100*
	Propan-1-ol	25.35^a^	9.77^b^	*−61.4*	22.74^a^	8.79^b^	*−61.4*	23.67^a^	4.40^c^	*−81.4*
	Ethyl Butanoate	0.10^a^	0.02^b^	*−79.3*	0.08^a^	0.02^b^	*−75.5*	0.09^a^	0.01^b^	*−86.8*
	2-Methylpropan-1-ol	23.59^a^	13.80^b^	*−41.5*	21.21^a^	12.37^b^	*−41.7*	22.38^a^	4.96^c^	*−77.8*
	3-Methylbutyl Acetate	3.60^a^	0.54^b^	*−85.0*	2.80^a^	0.49^b^	*−82.5*	3.33^a^	0.66^b^	*−80.2*
	3-Methyl-1-Butanol	146.92^a^	45.65^c^	*−68.9*	129.46^ab^	41.87^c^	*−67.7*	136.78^b^	37.20^c^	*−72.8*
	Ethyl Hexanoate	0.36^a^	0.03^b^	*−92.2*	0.26^a^	0.02^b^	*−92.2*	0.29^a^	0.00^b^	*−100*
	Ethyl Octanoate	0.34^a^	0.01^c^	*−96.1*	0.13^b^	0.00^c^	*−100*	0.14^b^	0.01^c^	*−90.4*

**B**										
Measurements		Trial 1			Trial 2			Trial 3		
		Standard Beer (S1)	Dealcoholized Beer (D1)	% Change	Standard Beer (S2)	Dealcoholized Beer (D2)	% Change	Standard Beer (S3)	Dealcoholized Beer (D3)	% Change
Ethanol Concentration (ABV)		4.31^a^	0.35^d^	*−91.9*	4.29^c^	0.35^d^	*−91.8*	4.30^b^	0.08^e^	*−98.1*
pH		4.09^b^	4.17^a^	*+2.0*	4.08^b^	4.15^a^	*+1.7*	4.09^b^	4.16^a^	*+1.7*
Bitterness Units		23.71^a^	17.03^b^	*−28.2*	24.13^a^	15.58^b^	*−35.4*	23.93^a^	15.12^b^	*−36.8*
Total Polyphenols (mg/L)		381.66^a^	258.94^bc^	*−32.2*	392.87^a^	243.63^c^	*−38.0*	340.94^ab^	237.16^c^	*−30.4*
Volatile Compounds (mg/L)	Acetaldehyde	3.11^bc^	3.42^b^	*+9.7*	3.95^ab^	1.96^c^	*−50.3*	3.52^ab^	4.64^a^	*+31.8*
	Ethyl Acetate	19.32^a^	3.83^b^	*−80.2*	19.93^a^	3.46^b^	*−82.6*	20.48^a^	1.11^c^	*−94.6*
	2-Methylpropyl Ethanoate	0.00^b^	0.01^a^	*+100*	0.01^ab^	0.01^ab^	*0*	0.00^b^	0.00^b^	*0*
	Propan-1-ol	62.84^a^	9.77^b^	*−84.4*	61.95^a^	8.79^b^	*−85.8*	66.30^a^	2.13^c^	*−96.8*
	Ethyl Butanoate	0.07^a^	0.02^b^	*−72.7*	0.08^a^	0.02^b^	*−73.3*	0.07^a^	0.00^c^	*−100*
	2-Methylpropan-1-ol	50.87^a^	13.80^b^	*−72.9*	50.30^a^	12.37^b^	*−75.4*	50.97^a^	3.65^c^	*−92.8*
	3-Methylbutyl Acetate	1.54^a^	0.54^b^	*−65.1*	1.58^a^	0.49^b^	*−68.9*	1.50^a^	0.19^c^	*−87.5*
	3-Methyl-1-Butanol	146.80^a^	45.65^b^	*−68.9*	141.30^a^	41.87^b^	*−70.4*	143.80^a^	16.19^c^	*−88.7*
	Ethyl Hexanoate	0.26^a^	0.03^b^	*−89.2*	0.26^a^	0.02^b^	*−92.3*	0.23^a^	0.00^b^	*−100*
	Ethyl Octanoate	0.21^b^	0.01^c^	*−93.5*	0.26^a^	0.00^c^	*−100*	0.20^b^	0.00^c^	*−99.2*

Further analysis was performed using SPME-GC–MS and LE-GC–MS to understand reductions of compounds not found through HS-GC-FID (shown in Table 3). Most compounds were found to significantly decrease after dealcoholization for both lager and stout trials, showing that it is not just ethanol that is removed when using RO membranes. However, differences between replicate trials for each beer were found suggesting a lack of consistency. Some compounds were also found to increase after dealcoholization.

**Table 3:** LogP values and molecular weight of compounds detected by SPME-GC–MS and LE-GC–MS, with % change for each beer style replicate dealcoholisation trial calculated from original beer peak area minus dealcoholized beer peak area. LogP values found through *EPI* Suite™ (4.11, U.S Environmental Protection Agency, Washington, USA). Identification method also included (AS = authentic standard, N = mass spectrum compared to NIST database). Compounds which increased after dealcoholisation are shown in green.

PLS-R was used as an attempt to model the relationship between volatile compounds, molecular descriptors and their removal from the beer. Of the 105 molecular descriptors explored, three main molecular descriptors were found, which can be used to explain the pathway of certain molecules through the membrane. The same descriptors were found for both beer styles, which included one surface area, volume and shape descriptor (*pmiZ)* and two subdivided surface areas descriptors (*SlogP_VSA3, SMR_VSA7)* (for more information on the molecular descriptors see Appendix Table 2)*.*

### 3.3 Sensory results

The mean attribute scores and results from ANOVA and Tukey’s HSD for the twenty-four aroma, flavour, taste and mouthfeel attributes for the NAB and full strength lagers using QDA with the trained panel were calculated.

#### 3.3.1 Lager

**Fig. 2:**
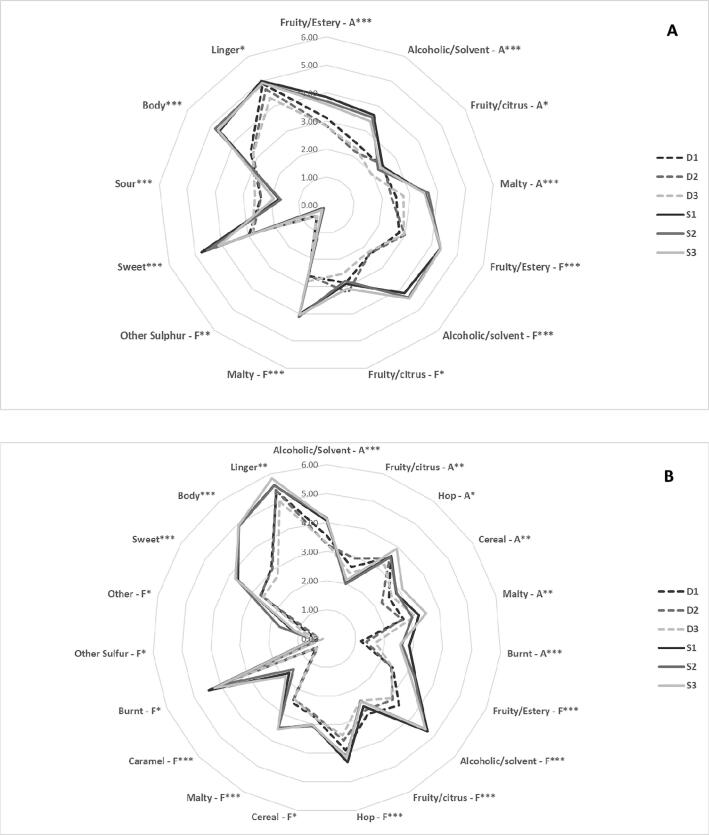
Spider plot of mean significant sensory attribute intensities from QDA trained panel data for (A) Lager (B) Stout. Terms with ‘– A’ after are aroma, and terms as ‘– F’ are flavour attributes. Terms with *** are significantly different between products at p < 0.0001; **p < 0.01, *p < 0.05.

ANOVA revealed differences for ‘fruity/estery aroma’, ‘alcoholic/solvent aroma’, ‘fruity/citrus aroma’, ‘malty aroma’, ‘fruity/estery flavour’, ‘alcoholic/solvent flavour’, ‘fruity/citrus flavour’, ‘malty flavour’, ‘other sulfur flavour’, ‘sweet’ and ‘sour’ tastes, ‘linger’ aftertaste and ‘body’ attributes (p < 0.0001). A spider plot (Fig. 2a), shows average ratings and significant sensory attribute terms for each trial of both standard and dealcoholized samples. Samples S1, S2 and S3 were found to be significantly higher (p < 0.0001) for the attributes ‘fruity/estery aroma’, ‘alcoholic/solvent aroma’ and ‘malty aroma’, ‘fruity/estery flavour’, ‘alcoholic/solvent flavour’ and ‘malty flavour’, ‘sweet’ and ‘body’ compared to the dealcoholized samples (D1, D2, D3). However, for samples D1, D2 and D3 ‘sour’ was significantly higher (p < 0.0001). The attributes ‘fruity/citrus aroma’ and ‘fruity/citrus flavour’ and ‘linger’ showed no significant difference between the standard and dealcoholized samples, however differences between dealcoholized samples were discovered, with trial D3 having significantly lower amounts of ‘fruity/citrus aroma’ and ‘fruity/citrus flavour’ compared to trials D1 and D2.

#### 3.3.2 Stout

For the stout, ANOVA revealed significant differences for ‘alcoholic/solvent aroma’, ‘fruity/citrus aroma’, ‘hop aroma’, ‘cereal aroma’, ‘malty aroma’ and ‘burnt aroma’, ‘alcoholic/solvent flavour’, ‘fruity/citrus flavour’, ‘hop flavour’, ‘cereal flavour’, ‘malty flavour’, ‘burnt flavour’, ‘caramel flavour’, ‘other sulfur flavour’ and ‘other flavours’, ‘sweet’ taste, ‘linger’ aftertaste and ‘body’ attributes. Fig. 2b shows that samples S1, S2 and S3 were significantly higher (p < 0.0001) for the attributes ‘alcoholic/solvent aroma’, ‘burnt aroma’, ‘alcoholic/solvent flavour’, ‘fruity/estery flavour’, ‘fruity/citrus flavour’, ‘hop flavour’, ‘malty flavour’, ‘caramel flavour’, ‘sweet’ and ‘body’ than D1, D2 and D3. However, for samples D1, D2 and D3 ‘fruity/citrus aroma’ was significantly higher (p < 0.0001). Differences between dealcoholized samples were also shown, with higher levels of ‘cereal aroma’, ‘malty aroma’ and ‘burnt aroma’ and decreased ‘linger’ in trial D3 compared to D1 and D2.

### 3.4 Correlation between physicochemical and sensory results

#### 3.4.1 Lager

**Fig. 3:**
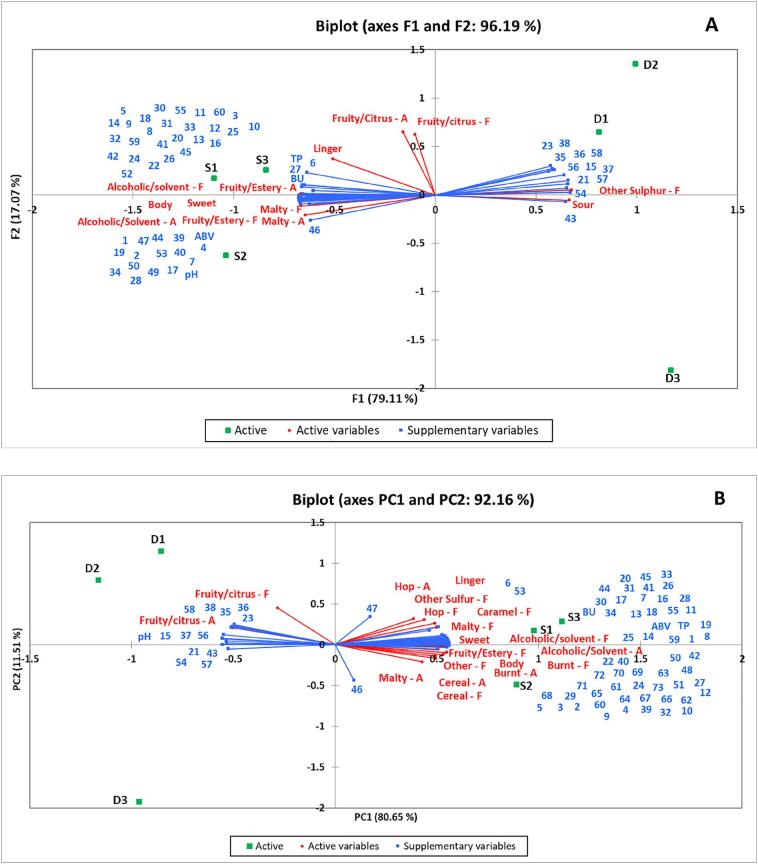
Principal component analysis (PCA) biplot of significant physicochemical properties and sensory attributes present on principle component 1 and 2 by the covariance of means across A) Lager and B) Stout samples. Green shows the 6 samples analysed, with sensory attributes shown in red and physicochemical properties in blue. The numbers in blue correspond with the volatile compound numbers shown in brackets in Table 3. (For interpretation of the references to colour in this figure legend, the reader is referred to the web version of this article.)

All significant physicochemical and sensory results were used to create a PCA plot (Fig. 3a). The first two principal components (PCs) of the model accounted for 96.19% of variation in the data, with most of the variance (79.11%) explained by the first principal component (PC1). PC1 was positively correlated with the sensory attributes ‘other sulfur flavour’ (0.975) and ‘sour’ (0.980), and compounds 2-(4-methylcyclohex-3-en-1-yl)propan-2-ol (0.884), methyl decanoate (0.963), 3,7-dimethyl-1,6-octadien-3-ol (0.855), methyl octanoate (0.971), 2-methylbutanoic acid (0.955), benzene (0.989), 1,7,7-trimethylbicyclo[2.2.1]heptan-2-one (0.871), terpin-4-ol (0.855), 1-methyl-4-(propan-2-yl)benzene (0.943) and (1R,4S,6S)-4,7,7-trimethylbicyclo[4.1.0]hept-2-ene (0.986). PC1 was negatively correlated with all other sensory attributes and physicochemical properties (all attributes and properties < −0.749). PC2 (showing 17.07% variation in data) was strongly positively correlated with ‘fruity/citrus aroma’ (0.961) and ‘fruity/citrus flavour’ (0.915). A significant difference between the first two trials (D1 and D2) compared to the third trial (D3) was clearly shown, with sample D3 positioned in the lower quadrant.

#### 3.4.2 Stout

The PCA for stout samples (Fig. 3b) showed again most of the variation in the first two PCs (92.16%). PC1 (80.65%) was strongly positively correlated with nearly all sensory attributes and physicochemical properties (>0.900), apart from ‘fruity/citrus aroma’ (−0.895) and pH (−0.949), 2-(4-methylcyclohex-3-en-1-yl)propan-2-ol (−0.881), methyl decanoate (−0.951), 3,7-dimethyl-1,6-octadien-3-ol (−0.892), methyl octanoate (−0.987), 1,3,3-trimethyl-2-oxabicyclo[2.2.2]octane (−0.861), 2-methylbutanoic acid (−0.935), benzene (−0.989), 1,7,7-trimethylbicyclo[2.2.1]heptan-2-one (−0.907), terpin-4-ol (−0.893), 1-methyl-4-(propan-2-yl)benzene (−0.974) and (1R,4S,6S)-4,7,7-trimethylbicyclo[4.1.0]hept-2-ene (−0.984) which were negatively correlated. PC2 (11.51%) was strongly correlated with ‘fruity/citrus flavour’ (0.804) and negatively correlated with acetaldehyde (−0.775). As with the lager, a significant difference between D1 and D2 compared to D3 was shown.

## 4 Discussion

### 4.1 Key physicochemical and sensorial changes of dealcoholized beers as a result of reverse osmosis

For the first time an understanding of the impact of RO on the physicochemical and sensorial properties of NAB is discussed in detail, by comparing the standard beers and their dealcoholized counterparts. Overall, data clearly showed that there were key volatile losses for both beer styles resulting in changes to the sensory profile.

Although ethanol was removed by a minimum of 91% in the present study, there was also a significant reduction in many other important physiochemical beer properties, including bitterness units, total polyphenols and key esters and higher alcohols. Other compounds such as carboxylic acids and aldehydes were also significantly reduced. This agreed with previous research conducted using very similar techniques to dealcoholize a starting beer of 4.9% v/v to 1.0% v/v, which resulted in large losses of volatiles (77% total esters, 68% total higher alcohols) (Kavanagh, Clarke, Gee, Miles, & Nicholson, 1991). These results however, seemed to be lower than the present study, but this could have been attributed to a lower beer feed temperature (5 °C), which may have reduced volatile losses (Alcantara et al., 2016).

It has previously been hypothesised that compounds with a similar structure and molecular weight to ethanol would be removed during membrane dealcoholization, whilst anything more complex would be retained (Ben-David et al., 2006, Catarino et al., 2006, Falkenberg, 2014, Schutte, 2003). All compounds detected in this study had a higher molecular weight than ethanol and therefore theoretically should have been rejected by the membrane, yet some were removed by up to 100%. When considering esters and higher alcohols, removal appeared to increase with increasing size (e.g. ethyl acetate up to ethyl decanoate), contradicting this hypothesis and results from previous reported studies. No trend in terms of Log*P* values, a measure of polarity of the compound, were found to explain this. Key smaller esters and higher alcohols present in beer (3-methylbutyl acetate, 2-methylpropyl acetate, 3-methyl-1-butanol and 2-methylpropan-1-ol) were found to be the compounds with the highest retention, and this was believed to be due to the additional methyl group within these molecules increasing branching, as well as decreased solubility in water (Falkenberg, 2014, Schutte, 2003). Overall, it appeared that compounds removed at a high level were relatively linear molecules, with low levels of branching. Other compounds which had increased branching or the presence of a benzene ring were retained, suggesting that chemical structure was important. This was confirmed using PLS-R analysis with MOE, showing that surface area + volume + shape are the key drivers of the effect, a factor which has not been used to explain this phenomenon before. By proposing a mechanism as to why certain compounds were reduced, research can be conducted in further studies on different membrane composition or post-production processes to rectify this problem. Suggestions for further work in this area are to confirm this mechanism in more detail, by selecting key marker compounds with different structural properties and spiking them into the beer before RO dealcoholisation. Confirming the proposed mechanism will help to direct post production methods to enhance compounds that have been fully removed using RO techniques, which have recently been found to be important in altering the sensorial profiles of the final NAB (Ramsey et al., 2021).

In addition, for the first time the effect of RO on sensorial properties of beer is reported. Ethanol has been found in previous research to enhance the perception of fruity flavour, alcoholic/solvent, sweetness and fullness/body (Clark et al., 2011, Langstaff et al., 1991, Martin and Pangborn, 1970, Ramsey et al., 2018, Williams and Rosser, 1981), with previous research also showing that RO removes volatiles that contribute to these attributes (e.g esters contributing to fruity flavour) (Alcantara et al., 2016, Catarino et al., 2007, Kavanagh et al., 1991), thus the significant attributes found in the present study confirm these findings. ‘Malty aroma’ and ‘malty flavour’ were also found to be significantly higher in the standard beers here, which has previously been found to be the dominant attribute in regular beers before swallowing (Missbach et al., 2017), as well as a driver of consumer liking in combination with the attribute ‘sweet’ (Porretta and Donadini, 2008, Ramsey et al., 2018). Sensory perceptions of ‘body’ were also found to be significantly lower in the dealcoholized samples suggesting that mouthfeel enhancers, such as sugars, were removed by the membrane due to their molecular size (Müller et al., 2017).

Overall, it was clear that the standard sample had higher amounts of volatile flavour compounds and sensory attributes compared to the dealcoholized, showing that there are extreme losses when subjecting a beer to RO. Suggestions to improve the final product and increase its comparability with the standard beer therefore include; altering the brewing process to account for volatile aroma losses, using special yeasts which can produce higher levels of higher alcohols and esters during fermentation, changing the composition of brewing raw materials or selecting an RO membrane with a different composition.

### 4.2 Matrix-membrane interactions

Overall, it was shown that RO removed key components of beer, but some differences were found between beer styles. The sensory data showed the dealcoholized lagers were perceived to be significantly more ‘sour’ and have increased ‘sulfur flavour’ compared to the standard lager, yet these attributes were not found to be significantly different for the stouts. Previous research suggested that physical dealcoholization techniques can produce a beer that is unbalanced in flavour, with significant increased perceived acidity due to removal of key esters and higher alcohols (Müller et al., 2017), denoting why the dealcoholized lager may have been perceived as more sour here. The perceived increase of sulfur flavours within the dealcoholized lagers could also simply be due to the lack of other flavours which normally work synergistically to cover up such ‘off-flavours’ (Kaipainen, 1992), yet this may not have been shown in the stout due to increased amounts of other flavour compounds (such as pyrazines and furans). No volatile compounds were identified to correlate to the attribute of ‘sulfur flavour’, but it is believed this may have been due to the increased presence of highly odour active compounds at very low concentrations within the dealcoholized beers, which were not discovered in GC–MS analysis. This could include sulfur compounds relevant in beer including: dimethyl sulfide (DMS), dimethyl disulfide, dimethyl trisulfide and sulfur dioxide.

In addition, stout trials took significantly longer to dealcoholize due to a slower flow rate through the membrane. It is considered that this could have been due to the starting raw materials of the stout, which contained five different malts, as well as flaked oats and roasted barley adjuncts. These previously have been found to clog membranes due to residual high molecular weight β-glucans (Briggs, Boulton, Brookes, & Stevens, 2004). Therefore suggestions could be made to select beers for membrane filtration made without adjuncts, to ensure less membrane clogging, quicker processing times, as well as lower production costs (Falkenberg, 2014). However, should technological advances solve the issue with processing times associated with high residual molecular weight β-glucans, then using RO techniques for beer matrices with increased levels of branched flavour compounds (such as the stout here) may result in less sensory and physicochemical changes in comparison to beers with linear flavour compounds (such as the lager here). Suggestions for further research are recommended to understand the effect of RO on other beer styles such as wheat beers and ales to confirm this.

### 4.3 Membrane efficiency

RO membranes can be expensive to purchase and therefore understanding their capabilities and efficiency is important for breweries. This is assessed by the quality and consistency of the finished product through replicate trials, as well as understanding indicators showing that the membrane may need to be replaced. Here three replicate trials were conducted for each beer style to further understand this.

Trial D3 for both beer styles showed differing physicochemical and sensory results compared to the two previous trials (D1 and D2) and was positioned separately on the PCAs. It appeared that more volatile losses occurred for trial D3 in both beer styles, with changes in sensory properties including decreased levels of ‘cereal’, ‘malty’ and ‘burnt’ aromas in D3 for the stout. This could however, have been due to the different starting volume of the stout (18L for D3, 50L for D1 and D2) influencing the differences in sensory attributes. Previous research discussed these changes to be due to a loss of selectivity within the membrane, indicating clogging of membrane pores, fouling or membrane cake build up (Falkenberg, 2014). Here it is believed that there was severe fouling of the membrane, meaning that certain compounds caused a blockage of the membrane pores making it difficult for ethanol to pass through into the permeate during trials, thus slowing down flow rates. Previous research has also assessed fouling coefficients of an RO membrane in a stout style beer using different diafiltration procedures (continuous and discontinuous), and found that diluting beer before dealcoholization, rather than after, could reduce fouling by almost half (Alcantara et al., 2016). It should be highlighted however, that this previous study was only assessed in lab-scale settings with smaller starting quantities of beer (500 mL) and therefore a suggestion for further work is to understand whether the same effect is shown with larger volumes of beer using a pilot-scale dealcoholization unit, similar to that used in the current study.

In addition, during physicochemical analysis, an increased amount of some volatile compounds were discovered in all dealcoholized samples compared to the standard beers. The discovery of this taint was unusual, as the starting beers either contained a very low level of these compounds or none at all. These compounds included certain terpenes and higher alcohols (4,7,7-trimethylbicyclo[4.1.0]hept-2-ene, 1,7,7-trimethylbicyclo[2.2.1]heptan-2-one, 2-(4-methylcyclohex-3-en-1-yl)propan-2-ol, 3,7-dimethyl-1,6-octadien-3-ol), with similar molecular weights (154.25 g/mol). The presence of these compounds also became apparent in the sensory results, with higher ratings for ‘fruity/citrus aroma’ and ‘fruity/citrus flavour’ in the dealcoholized samples for both beer styles. Panellists described this in additional comments as ‘ginger/orange/herbal/citrus’. It should be noted here, that the presence of this taint did not seem to effect other sensory results, with small differences still discovered between the dealcoholised and standard beers. Delving deeper into the results, it was clear to see that this phenomenon was limited to a small group of volatile compounds, which all had a similar cyclic structure. It is therefore believed that these could have been adsorbed within the membrane during preceding projects, where products known to contain some of these compounds were dealcoholized using the same membrane and system. Previous research also found similar results, with linear compound structures having an easier passage as they were more permeable to the membrane, whereas cyclic structures entered the membrane during cross filtration and then became stuck (Falkenberg, 2014). It is believed that this taint was therefore part of a contamination residue on or within the membrane, with these compounds being pulled through into the beer when dealcoholization took place. Consequently, D3 was a ‘cleaner’ replicate, as most of the contamination residue from the taint had been removed during the first two trials. This was revealed in the sensory data for the lager, as trial D3 had significantly lower amounts of ‘fruity/citrus aroma’ and ‘fruity/citrus flavour’ compared to trials D1 and D2. With this residue being removed in D3 however, it also meant that more of the key volatiles could be removed from the beer making their way through to the permeate as ‘waste’, which was shown by increased losses of volatiles in this replicate. Again, this could however, have also been down to the different starting volumes for D3 lager and stout (44.2 L for lager and only 18 L for stout), meaning that less processing time was needed. Many of these compounds were insoluble in water and therefore cleaning with water and NaOH (as suggested by the membrane supplier) may not have removed all traces. Therefore, the importance of thorough cleaning of all kit is highlighted here. In addition, it is recommended that a separate membrane be used for different starting product matrices.

Overall, this research showed that using RO as a membrane filtration technique can produce a NAB with reduced physicochemical and sensory attributes compared to its standard alcohol counterpart. Further improvements to the process, as well as increased understanding of product matrix interactions, and RO membrane-matrix interactions are however still needed to produce a more acceptable NAB for consumers using this technique.

## 5 Conclusion

This study evaluated the impact of RO on the physicochemical and sensory properties of two different beer styles (lager and stout). Results showed that there was clear differentiation between a standard alcohol beer and its lower alcohol counterpart, with severe removal of numerous volatile compounds, including a 70% reduction in 3-methyl-1-butanol and 92% reduction in ethyl hexanoate resulting in a change in sensory properties. Dealcoholized beers had a decreased presence of the sensory attributes ‘fruity/estery aroma’, ‘alcoholic/solvent aroma’, ‘malty aroma’, ‘fruity/estery flavour’, ‘alcoholic/solvent flavour’, ‘malty flavour’, ‘sweetness’ and ‘body’. Removal of volatile compounds by the RO membrane was found to not be due to molecular size, but instead due to molecular structure with compounds with increased levels of branching (including 3-methylbutyl acetate and 3-methyl-1-butanol) retained to a higher degree in comparison with more linear structured compounds. This was confirmed using molecular operation environment descriptors, which showed that surface area + volume + shape were the key drivers of the effect. By proposing a mechanism as to why certain compounds were reduced, further studies can rectify this problem by exploring the effect of different membrane compositions or post-production processing techniques. The interactions between RO membranes and different product matrices were also reported, with more sensorial differences discovered between the lagers compared to the stout. This showed that dealcoholizing a lager may face increased challenges as the removal of volatiles leads to a lack of other flavours, which normally work synergistically to cover up ‘off-notes’ such as ‘sour’ taste and ‘sulfur flavour’. However, stouts present more of a challenge in terms of membrane clogging as they contain greater higher molecular weight compounds which have increased branching or ring structures. It was also noted that deep cleaning of the membrane between trials is required, as well as the use of separate membranes for different product matrices to avoid product contamination associated with membrane fouling resulting in taints.

This research is important for the international brewing industry as the global demand for NAB increases. RO as a technique to produce NABs is explored, furthering knowledge by reporting results from replicate trials, as well as results using different product starting matrices. This can help breweries understand what needs to be corrected when RO membranes are used to produce dealcoholized beers.

## CRediT authorship contribution statement

**Imogen Ramsey:** Conceptualization, Methodology, Formal analysis, Investigation, Writing - original draft. **Qian Yang:** Writing - review & editing, Supervision. **Ian Fisk:** Writing - review & editing, Supervision. **Charfedinne Ayed:** Formal analysis, Writing - review & editing. **Rebecca Ford:** Conceptualization, Methodology, Writing - review & editing, Supervision.

## Declaration of Competing Interest

The authors declare that they have no known competing financial interests or personal relationships that could have appeared to influence the work reported in this paper.
