# Supplementary material for: Assessing the sensory and physicochemical impact of reverse osmosis membrane technology to dealcoholize two different beer styles
Source: Food Chem X. 2021 Apr 30;10:100121. doi: 10.1016/j.fochx.2021.100121 (PMC8220173; doi:10.1016/j.fochx.2021.100121)
Supplement: Supplementary data 1 [file mmc1.doc]

# Supplementary Materials

***Appendix Table 1: Attributes, definitions and reference standards used in QDA trained sensory panel for lager and stout (n=10). Attributes with * are those used only for lagers and those with ** for attributes used only with stouts***

|  | Attribute | Description | Reference |
| --- | --- | --- | --- |
| Aroma/Flavour | Fruity/Estery | Esters derived from fermentation. Flavours including: strawberry, raspberry, peach, apricot, pineapple, banana, peardrops, candy sticks | 0.5-10mg 3-methylbutyl acetate/L beer (FlavorActiv™);  0.05-0.25mg ethyl hexanoate/L beer (FlavorActiv™) |
|  | Alcoholic/Solvent | Ethanol and higher alcohols from fermentation. Flavours including: Ethanolic, vinous, warming, raw, higher alcohols | Potable alcohol 0-9% ABV in beer |
|  | Fruity/Citrus | Citrus fruit character from hops. Flavours including : Grapefruit, lemon, lime, orange | Hop essential oils |
|  | Hop** | Fresh, resinous, herbal, grassy, spicy | 0.01-0.2mg spicy hop essential oils/L beer (FlavorActiv™) |
|  | Floral/Fragrant* | Floral character from hops. Flavours including: Geraniol, floral, fragrant, elderflower, perfumed | 0-100ug geraniol/L beer (FlavorActiv™) |
|  | Spicy/Grassy Hop* | Grassy character from hops. Flavours including : Freshly cut grass, resinous, herbal, grassy, spicy | 10-40ug eugenol/L beer (FlavorActiv™);  1 Cis-3-Hexenol capsule/ L beer (FlavorActiv™) |
|  | Cereal | Cereal character from grains. Flavours including: Cereal, grainy, hay, straw, worty, bran | 10ug 2-methyl propiondaldehyde/L beer (FlavorActiv™) |
|  | Malty | Malted cereal from grains. Flavours including: Malty, nutty, vanilla | Malt extract  Vanilla exctract |
|  | Caramel** | Caramel, nutty, fudge | 3-ethyl,2,5-dimethylpyrazine (FlavorActiv™) |
|  | Burnt** | Roasted, burnt, ashy | N/A |
|  | DMS | Dimethyl sulphide (part of the sulfury character of lagers). Flavours including: Sweetcorn, baked beans, tinned tomatoes | 10-150ug dimethyl sulfide /L beer (FlavorActiv™) |
|  | Other Sulfur | Any other sulfurs found in lager beers. Flavours including: Sulfidic (eggy), sulfitic (struck match), yeasty, bready, meaty, drainy, garlic, onion, cooked veg., lightstruck. | 5-125ug hydrogen sulphide/L beer (FlavorActiv™);  15mg sodium sulfite/L beer (FlavorActiv™);  0.05-0.3ug dimethyl trisulfide/L beer (FlavorActiv™);  2-15mg acetaldehyde/L beer (FlavorActiv™);  8-600ug diacetyl/L beer (FlavorActiv™);  30-200mg acetic acid/L beer (FlavorActiv™);  1 chlorophenol capsule/L beer (FlavorActiv™) |
| Taste | Sweet | Sweet taste from residual sugars. Flavours including: Sugar, saccharin, honey, syrup | Sucrose |
|  | Sour | Sour taste. Acidic, mouthpuckering | 90-300mg citric acid/L beer (FlavorActiv™) |
|  | Bitter | Bitter taste mostly from hop alpha iso acids. Tonic water, quinine | 5 Bitterness Units (BU) iso-α-acids/L beer (FlavorActiv™) |
| Mouthfeel | Astringent | Astringent mouthfeel, mainly from hop components. Tannic, drying, black tea | 1 Saponin capsule/L beer (FlavorActiv™) |
|  | Body | Mouthfeel, density, associated to non fermentable sugars, ethanol and higher alcohols. Thick, viscous, full, thin, watery | N/A |
|  | Linger | Length of flavour in mouth. Aftertaste, length, intensity | N/A |

***Appendix Table 2:*** *Main molecular descriptors discovered by PLS-R analysis and their definitions from MOE (2002.03, Chemical Computing Group, Montreal, Canada)*

| Molecular Descriptor | Definition |
| --- | --- |
| *pmiZ* | Spatial external 3D descriptor based on the z component of principal moment of the inertia. |
| *SlogP_VSA3* | Represents the Van der Vaals surface area of the atoms contributing to the logP (o/w) of the molecule in the range (0, 0.1). |
| *SMR_VSA7* | Sum of νi, such that Ri > 0.56. This is the subdivided surface area based on an approximate accessible van der Waals surface area (in Å^2^) calculation for each atom, ν_i_, along with some other atomic property, p_i_. |
